# Human nucleosomes: special role of CG dinucleotides and Alu-nucleosomes

**DOI:** 10.1186/1471-2164-12-273

**Published:** 2011-05-31

**Authors:** Thomas Bettecken, Zakharia M Frenkel, Edward N Trifonov

**Affiliations:** 1 CAGT-Center for Applied Genotyping, Max Planck Institute of Psychiatry, Kraepelinstr. 2-10, D-80804 Munich, Germany; 2 Genome Diversity Center, Institute of Evolution, University of Haifa, Mount Carmel, Haifa 31905, Israel; 3 Division of Functional Genomics and Proteomics, Faculty of Science, Masaryk University, Kotlarska 2, Brno CZ-61137, Czech Republic

## Abstract

**Background:**

The periodical occurrence of dinucleotides with a period of 10.4 bases now is undeniably a hallmark of nucleosome positioning. Whereas many eukaryotic genomes contain visible and even strong signals for periodic distribution of dinucleotides, the human genome is rather featureless in this respect. The exact sequence features in the human genome that govern the nucleosome positioning remain largely unknown.

**Results:**

When analyzing the human genome sequence with the positional autocorrelation method, we found that only the dinucleotide CG shows the 10.4 base periodicity, which is indicative of the presence of nucleosomes. There is a high occurrence of CG dinucleotides that are either 31 (10.4 × 3) or 62 (10.4 × 6) base pairs apart from one another - a sequence bias known to be characteristic of Alu-sequences. In a similar analysis with repetitive sequences removed, peaks of repeating CG motifs can be seen at positions 10, 21 and 31, the nearest integers of multiples of 10.4.

**Conclusions:**

Although the CG dinucleotides are dominant, other elements of the standard nucleosome positioning pattern are present in the human genome as well.

The positional autocorrelation analysis of the human genome demonstrates that the CG dinucleotide is, indeed, one visible element of the human nucleosome positioning pattern, which appears both in Alu sequences and in sequences without repeats. The dominant role that CG dinucleotides play in organizing human chromatin is to indicate the involvement of human nucleosomes in tuning the regulation of gene expression and chromatin structure, which is very likely due to cytosine-methylation/-demethylation in CG dinucleotides contained in the human nucleosomes. This is further confirmed by the positions of CG-periodical nucleosomes on Alu sequences. Alu repeats appear as monomers, dimers and trimers, harboring two to six nucleosomes in a run. Considering the exceptional role CG dinucleotides play in the nucleosome positioning, we hypothesize that Alu-nucleosomes, especially, those that form tightly positioned runs, could serve as "anchors" in organizing the chromatin in human cells.

## Background

The periodical distribution of various dinucleotides along eukaryotic DNA sequences with a period of 10-11 bases is commonly considered as the manifestation of a nucleosome positioning signal present in the sequences [1-8]. The period, the more accurate value of which is 10.4 bases [9-12], corresponds to the helical repeat of DNA in the nucleosome. The positioning signal in human nucleosomes is rather weak and lacks the periodical AA and TT dinucleotides [13], while in yeast and nematodes the periodical nucleosome signals are dominated by AA and TT dinucleotides [5,6]. However, RR and YY dinucleotides, GG and CC in particular, have been shown to contribute to the human nucleosome positioning signal [13,14]. Whole-genome calculations for 13 diverse eukaryotes [8] confirmed the exceptional lack of visible dinucleotide periodicities in the human genome, where only CG showed a signal. Nucleosomes on Alu sequences, which are known to contain strongly periodical CG dinucleotides, are apparently representatives of a special class.

## Methods

The full human genome sequence (build hg18) was copied from the UCSC genome server http://www.genome.ucsc.edu. The sequence had been assembled by the International Human Genome Project sequencing centers (March 2006). For filtering out repeats, the sequence data available under the label "masked" (hg18, file ChromFaMasked.zip, genome.ucsc.edu) was used.

All programs employed to calculate the DNA composition and derivation of the distance diagrams (autocorrelations) are either Perl scripts or C++ programs, both original. The auto-correlation was calculated as follows. For a dinucleotide MN at a given position, all distances to other MN dinucleotides downstream were counted and restricted to the size of the window. This was applied to all dinucleotide occurrences in the sequence. Essentially, the procedure scores all distances and reveals those which are preferred. The routine was disrupted when filtered repeat sequences or the end of a chromosome occurred within the window size limit. In order to avoid the end effect of the short-range distances in the positional correlation analysis, the last dinucleotides within the window size region at the sequence ends were excluded.

For the mapping of Alu sequences, the human Alu-Sx subfamily consensus sequence [15] was matched with the full human genome, using the software BLAST (release 2.2.21, taken from ftp://ftp.ncbi.nlm.nih.gov/blast/executables/) with standard (default) searching parameters. When starting positions of the matches were less than 250 bases apart, only the upstream copies were selected.

## Results and Discussion

### CG-periodicities in the human whole-genome sequence

**Figure 1:**
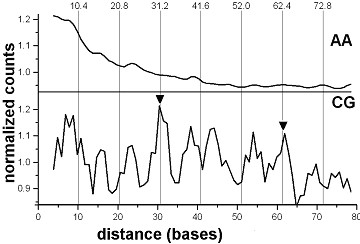
**AA and CG dinucleotides in the human genome**. Positional autocorrelation of AA and CG dinucleotides in the complete human genome. The normalized histograms of occurrences of the dinucleotide pairs at distances 2-80 bases from one another are shown. The histograms are smoothed by running average of 3 positions. Level 1.0 corresponds to average scores of the respective raw histograms (3.29 × 10^7 ^for AA and 6.38 × 10^5 ^for CG). Two peaks on the CG curve (arrowheads, at positions 31 and 62) correspond to 10.4 × 3 base distances between CG dinucleotides in Alu sequences (see text). Vertical grid lines indicate the 10.4 base nucleosome DNA period.

The whole-genome distance analysis of the human genome sequence reveals an obvious 10.4 base periodicity for the dinucleotide CG only, but not for any of the other dinucleotides. The autocorrelation functions for AA and CG are shown in Figure 1. CG dinucleotides do show distinct peaks of high relative amplitudes at distances 31 (10.4 × 3) and 62 (10.4 × 6), characteristic for Alu sequences [16]. The other peaks in the CG histogram are typically 8 bases apart, and correspond to the hidden 8 base periodicity of the Alu sequences (*ibid*.). In contrast, AA dinucleotides display no periodicity (Figure 1).

**Figure 2:**
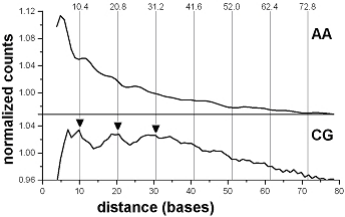
**AA and CG dinucleotides in the human genome (repeats masked)**. Positional autocorrelation of AA and CG dinucleotides in the human genome with repeating sequences masked. The plots are derived and treated as in Figure 1. The levels 1.0 correspond to normalized averages of the scores, within the interval 0-80 bases (1.13 × 10^7 ^for AA and 2.75 × 10^5 ^for CG). Three 10.4 × n maxima (at positions 10, 21 and 31) are indicated by arrows.

When Alu repeats as well as all other repeating sequences are removed from the genome, using the "masked" version of the human genome (see Methods), the high and sharp CG-peaks at positions 31 and 62 bases disappear. Instead, the broad peaks at positions 10, 21 and 31 (Figure 2) appear, at positions that are the nearest integers to multiples of 10.4 bases (e.g., 10.4, 20.8, 31.2). No other dinucleotide periodicities in the human genome sequence are detected this way, confirming the earlier result [8]. The CG-containing Alu-sequences and periodical CG dinucleotides in the non-repetitive bulk of human DNA seem to be the only signatures of nucleosome positioning in the human genome (Figures 1 and 2), which can be revealed by the positional autocorrelation analysis. A more advanced and powerful method of extraction of the nucleosome positioning pattern is the Shannon N-gram extension [17], recently introduced to chromatin studies [18]. It allowed derivation of both dominant (TAAAAATTTTTA) and CG-containing (CGGAAATTTCCG) nucleosome positioning patterns for the human genome. The latter one is identical to the pattern for *C. elegans *nucleosomes [7] and apparently, represents those CG-containing human nucleosomes which cause the unusual CG-periodicity in the non-repetitive regions of human DNA. Other elements of the above CG-containing pattern may appear periodical when the nucleosome DNA sequences are analyzed rather than whole genome sequences (work in progress).

### A possible chromatin organizing role of Alu sequences

A model chromatin built from weak nucleosomes would very likely be unstable, having a loose structure and allowing for nucleosome sliding to alternative positions. One possible arrangement to avoid such instabilities would be the introduction of a certain number of strong uniquely positioned "anchoring" nucleosomes. These would serve as chromatin organizers, thus limiting the freedom of sliding of the other nucleosomes in between. Such a hypothetical arrangement has been previously described as the "parking lot model" [19].

**Figure 3:**
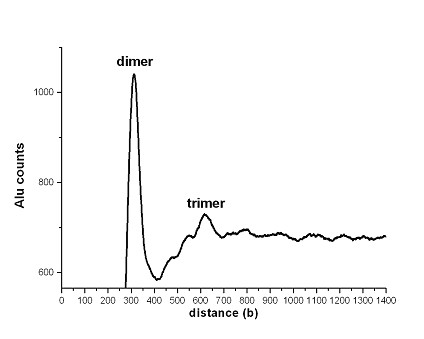
**Distances between Alu-repeats in the human genome**. Histogram presentation of distances between Alu-repeats (head to head).

The role of such hypothetical "anchors" in human chromatin may be played by the nucleosomes positioned on the Alu-sequences. The Alu-sequences contain the CG dinucleotides 31-32 bases apart, that is, at multiples of the nucleosome DNA period [16]. As periodical positioning of CG dinucleotides is an important component of the nucleosome positioning pattern [7], the Alu-sequences could be very well suited for nucleosome formation. Such nucleosomes are, indeed, observed experimentally [20,21]. Moreover, it has been demonstrated recently that the Alu-sequences have influence on the positioning of neighboring nucleosomes [22]. Size-wise, every Alu-sequence may harbor two nucleosomes. However, the Alu-sequences often appear also as tandem dimers and even trimers. In Figure 3, the histogram of distances between the Alu-repeats is shown. Two peaks are observed, at positions ~310 and ~620, corresponding to Alu-dimers and trimers, respectively. The tandem dimers (trimers) of Alu-sequences would contain four (six) nucleosomes each. Such "frozen" combinations of two, four or more tandem nucleosomes, additionally stabilized by their periodical arrangement, could presumably act as those hypothetical anchors. According to our calculations, the human genome contains a total of 1.16 million of such hypothetical Alu-anchors, of which 1,020,000 are singular repeats, 116,000 are dimers and 18,000 are trimers. This corresponds to an average spacer between the Alu sequences or Alu sequence clusters of about 2300 base pairs (tail to head), space enough to accommodate 10-15 nucleosomes. At this point, we would like to propose that repeat sequences in general may well have such a chromatin organizing function. The (19)_n _and (35)_n _tandem repeats of *C. elegans *[23], which contain the standard nucleosome positioning pattern AAATTTCCGG would be sites of formation of strong nucleosomes if our hypothesis holds. Tandemly repeating α-satellite sequences of primates carrying nucleosomes [24] and mouse 234-base satellite nucleosomes [25] could serve as two more examples of potential chromatin anchors.

### The dual role of CG dinucleotides

It was not until recently that evidence emerged on the role CG dinucleotides may have in nucleosome positioning. Their appearance in Alu-sequences at distances of multiples of 10.4 bases (31 or 32 bases) was the first indication of their phasing function [16]. Next, the analysis of the nucleosome DNA sequences of *C. elegans *showed that CG dinucleotides do have an unusually high positional preference within the 10-matrix of DNA bendability [7]. Finally, a spectacular 10.4 base periodicity of CG in the genome of *A. mellifera*, the honey bee, was discovered [8]. It turned out that the CG dinucleotide is, actually, among the strongest periodical elements (after AA and TT) in eukaryotic genomes.

The second obvious role of the CG dinucleotide is its potential to undergo C-methylation/-demethylation in many eukaryotic organisms. This modification is known to crucially impact gene expression and is leading to epigenetic phenomena [26,27]. It is known also, that the DNA methyltransferases preferentially target nucleosomes [28,29], so that the methylated CpGs are distributed with the period ~10 bases along the nucleosome DNA [29]. Nucleosomes containing CG dinucleotides in key positions for the nucleosome stability - in the minor grooves at the interface DNA/histones [7,30] - could be called epigenetic nucleosomes [16]. The C-methylation in CG dinucleotides may tune the stability of the nucleosomes in promoter regions [27], and modulate the stability of the proposed anchor nucleosomes, e. g., Alu-nucleosomes, containing many CG dinucleotides. In [31] it is demonstrated for the first time that CpG methylation renders compactness to nucleosomes, with DNA bound more tightly to the histone octamers.

### Are the weak nucleosomes phased?

The poor manifestation of dinucleotide periodicities in the human genome, namely CG only, suggests that the majority of human nucleosomes are rather weak. This means that there is only weak pressure by the nucleosome positioning signal. As experiment [32] and calculations (Gabdank *et al.*, unpublished data) show, even in the highly periodical genome of *C. elegans*, the majority of the nucleosomes are as weak as the "nucleosomes" mapped on random sequences. The mouse genome in which no dinucleotide periodicity is emerging with autocorrelation calculations [8], is especially interesting in this respect. This is a nightmare case for signal hunters, although there definitely must be a certain sequence specificity for chromatin organization. After all, the DNA in the mouse genome is packed into nucleosomes as well, and the mouse chromatin is not known to be any different from typical mammalian chromatin. It would be incorrect though, to conclude that weak nucleosomes are randomly distributed along the sequences. Let us consider a hypothetical natural sequence in which the positioning signal is not introduced. In that sense, the sequence would be "random". But the histone octamers would still bind to those segments of the sequence that do have some resemblance to the standard positioning pattern. They will form *weak *nucleosomes at *specific *positions along the sequence. The non-randomness of nucleosome positioning in natural genomes is evidenced by the existence of the "nucleosome repeat lengths" [33], from 160 to 240 bases, depending on the species.

## Conclusions

For detection of the periodical repetition of the DNA bendability pattern, whole-genome sequences with very weak or invisible periodicities are not suitable. The periodical signal extraction will probably succeed when it is applied instead to the comprehensive nucleosome DNA database sequences (work in progress). Due to the affinity of histone octamers to the segments with highest bendability, the sequences of the databases will contain the signal. For its extraction, the signal regeneration procedure can be used as described in [7]. This study and others [18,34] show that, no matter how weak the nucleosome positioning signal is, it can be traced and even characterized by one or another signal processing technique. It also shows that due to apparently species-specific sequence preferences, various different components of the general nucleosome positioning pattern can be used by different organisms. The preferential use of CG dinucleotides in human chromatin is the illustration. At the same time, since the physics of nucleosome positioning should be the same everywhere, the same universal pattern [35] should be used by all species. This does not exclude though, that there can be species-specific biases towards this or other selections of dinucleotides predominantly used for positioning of nucleosomes [8]. Finally, with the identification of nucleosome positioning CG and other dinucleotides, it seems very natural to extend these considerations to the variable sites in the eukaryotic and (especially) the human genome. Single nucleotide polymorphisms (SNPs), SNP haplotypes, repetitive sequences, whether stable or subject to expansion or contraction, appear in a new light, as respective nucleosomes involved may vary in strength and/or position.

## Authors' contributions

TB initiated the work, authored code, did part of the calculations and analyses and edited the manuscript. ZMF authored code, did part of the calculations and analyses and helped to draft the manuscript. ENT conceived the study, did part of the analyses, and drafted the manuscript. All authors read and approved the manuscript.
